# Depressive and Post-Traumatic Stress Symptoms and Their Associations with Health-Related Quality of Life in Women with Breast Cancer: A Cross-Sectional Secondary Analysis

**DOI:** 10.3390/medsci14060625

**Published:** 2026-09-30

**Authors:** Anastasia Karagiannaki, Vasiliki Michou, Evangelia Antoniou, Menelaos Zafrakas, Panagiotis Eskitzis

**Affiliations:** 1 Department of Midwifery, School of Healthcare Sciences, University of Western Macedonia, Keptse, 50200 Ptolemaida, Greece; anastasiakaragiannaki@hotmail.com (A.K.); peskitzis@uowm.gr (P.E.); 2 Department of Nursing, School of Health Sciences, International Hellenic University, 57400 Thessaloniki, Greece; 3 Department of Midwifery, University of West Attica, 12243 Athens, Greece; lilanton@uniwa.gr; 4 School of Health Science, International Hellenic University, 57400 Thessaloniki, Greece

**Keywords:** breast cancer, quality of life, depression, post-traumatic stress disorder

## Abstract

Background: Breast cancer (BC) significantly affects patients’ quality of life (QoL), not only through the physical burden of disease and its treatment but also through psychological mechanisms. The aim of this study was to investigate the associations between HRQoL, depressive symptoms, and post-traumatic stress symptoms in Greek women with BC, and to examine sociodemographic and clinical factors associated with depressive and post-traumatic stress symptom severity. Methods: This cross-sectional secondary analysis included 107 women with BC from a previously described cohort. Participants completed a structured demographic and clinical questionnaire, the Posttraumatic Stress Disorder Checklist for DSM-5 (PCL-5), the Patient Health Questionnaire-9 (PHQ-9), and the EORTC Quality of Life Questionnaire-Core 30 (EORTC QLQ-C30). Results: In total, 64.5% of participants had moderate to severe depressive symptoms, with a mean PHQ-9 score of 11.9. In exploratory adjusted analyses, mastectomy and an interval of 1–3 years since BC diagnosis were associated with higher PHQ-9 scores, whereas previous endocrine therapy alone was associated with lower PHQ-9 scores. Regarding post-traumatic stress symptoms, 51.4% of participants met all four PCL-5 DSM-5 symptom-cluster requirements for probable PTSD. Rural residence, mastectomy, and an interval of 1–3 years since BC diagnosis were independently associated with higher PCL-5 scores. Greater depressive symptom severity and probable PTSD status were both significantly associated with poorer HRQoL across multiple functional and symptom domains. Participants meeting the PCL-5 symptom-cluster criteria for probable PTSD also reported greater depressive symptom severity. Conclusions: Greek women with BC experienced a substantial burden of depressive and post-traumatic stress symptoms, which were closely interrelated and associated with poorer HRQoL. Exploratory multivariable analyses suggested associations between selected sociodemographic and clinical characteristics and psychological symptom severity; however, larger prospective studies are needed to confirm these findings. The findings support the relevance of psychological assessment as part of comprehensive BC care.

## 1. Introduction

Global cancer prevalence is projected to rise by approximately 47% by 2040 compared to levels reported in 2020 [1]. Breast cancer (BC) represents a significant global health burden, exerting extensive physical, emotional, and practical effects. It accounts for approximately 2.3 million new diagnoses annually and contributes to about 7% of all cancer-related mortality. Globally, it constitutes roughly one in four cancer cases and one in six cancer deaths, making it the most frequently diagnosed cancer in both high- and middle-income countries [1]. Advances in treatment modalities and improvements in healthcare systems have led to increased survival rates and a gradual decline in mortality. Consequently, there is a growing emphasis on addressing the long-term consequences of BC and its treatment, particularly the psychological and physical challenges faced by survivors [2].

BC exerts a profound impact on patients’ quality of life (QoL), not only through the physical burden of the disease and its treatment, but also via significant psychological consequences, including depression [3] and post-traumatic stress disorder (PTSD) [4]. Numerous studies have demonstrated that women diagnosed with BC frequently experience diminished health-related quality of life (HRQoL) across multiple domains, particularly during and after treatment [5,6,7,8,9]. A substantial body of evidence highlights the persistent impact of depression on BC survivors, with depressive symptoms commonly extending well beyond the treatment phase.

A substantial body of evidence highlights the psychological burden experienced by women with BC, with depressive symptoms potentially extending beyond the active treatment phase. Studies have shown that a significant proportion of women report symptoms of depression and anxiety within the first year after BC diagnosis, with psychological distress persisting in a subset of patients during subsequent years [10,11,12,13]. Importantly, longitudinal evidence comparing depressive symptoms before and after BC diagnosis has demonstrated a significant increase following diagnosis, particularly during the first year, compared with pre-cancer levels [14]. Furthermore, time since diagnosis or surgical intervention has been associated with variations in depression prevalence, with symptoms generally declining as survivorship progresses [15,16]. Epidemiological data indicate that between 22% and 38% of individuals diagnosed with BC exhibit symptoms of depression, with approximately 22% experiencing moderate to severe levels of depressive severity. With advances in treatment contributing to increased survival rates and prolonged life expectancy, the relevance of addressing mental health and overall QoL in BC survivorship has become increasingly important [16].

PTSD and post-traumatic stress symptoms have been recognized as clinically relevant psychological outcomes in oncological settings, including among patients with BC. The diagnostic and therapeutic processes associated with BC entail substantial psychological and physiological stressors that may extend beyond active treatment. These stress responses may manifest as persistent psychological distress, including fear of cancer recurrence and, in some patients, symptoms consistent with PTSD [17]. Meta-analytic findings estimate that approximately 9.6% of BC patients exhibit PTSD symptoms [18]. This estimate appears lower than rates of clinically significant post-traumatic stress symptoms reported in some other cancer populations; for example, meta-analytic evidence has indicated rates of approximately 17.7% among patients with head and neck cancer and 17.9% among adults with hematological malignancies [19,20]. However, compare these estimates cautiously because studies differ substantially in assessment instruments, diagnostic criteria, clinical characteristics, and timing of evaluation [18,19,20]. Notably, early investigations into this phenomenon, such as one of the first studies assessing QoL in relation to PTSD-like symptoms in BC survivors, reported a PTSD prevalence of 5–10% and found a strong inverse relationship between PTSD symptoms and QoL [21]. Further supporting this link, a large study of long-term BC survivors reported a PTSD prevalence of approximately 12% and found a significant association between post-traumatic stress and impaired QoL, supporting the clinical relevance of identifying affected patients despite the relatively modest overall prevalence [22].

Based on the above, the dynamic interplay between depression, PTSD, and QoL in BC survivors is of notable clinical relevance. Although the individual effects of depression and PTSD on QoL in BC patients have been documented (with PTSD significantly less common than depression), evidence regarding their combined or interactive impact on QoL outcomes is limited. Amir et al. [4] were among the first to assess the combined impact of PTSD symptoms and emotional distress on QoL in long-term survivors, demonstrating that both were significantly associated with reduced QoL across multiple domains. To the best of our knowledge, there are no studies evaluating the combined impact of PTSD and depression on QoL of BC patients in Greece. To date most studies have either focused on depression and anxiety or on QoL separately, without systematically evaluating PTSD symptoms [23,24]. These findings underscore the importance of ongoing psychological assessment and support in survivorship care. Thus, this cross-sectional secondary analysis aimed to provide additional psychological characterization of a previously described cohort of Greek women with BC by examining depressive and post-traumatic stress symptoms, their associations with HRQoL, and sociodemographic and clinical factors associated with psychological symptom severity.

## 2. Materials and Methods

### 2.1. Participants

Women diagnosed with BC who voluntarily expressed interest in participating were screened for eligibility based on predefined inclusion and exclusion criteria. Inclusion criteria were age ≥ 18 years, a confirmed diagnosis of BC, and current receipt of chemotherapy for BC. Exclusion criteria included patients who had not yet started BC treatment, had previously participated in a similar study, or were unwilling to provide the data required for study participation. Recruitment took place from May to October 2023.

### 2.2. Study Design

The present study is a cross-sectional secondary analysis of a previously described cohort of women with BC treated at Hippokration General Hospital, Greece. Women with BC were invited to participate in the study. Those who voluntarily agreed to take part were asked to complete four questionnaires: (1) a general questionnaire designed to collect BC-related demographic and clinical information, (2) the Post-traumatic Stress Disorder Checklist for DSM-5 (PCL-5), (3) the Patient Health Questionnaire-9 (PHQ-9), and (4) the EORTC Quality of Life Questionnaire-Core 30 (EORTC QLQ-C30). Participants in the present study comprised the same cohort of 107 women with BC previously described in our published study on HRQoL [25]. The previous publication focused primarily on HRQoL and its associations with sociodemographic and clinical characteristics using the EORTC QLQ-C30 [25]. Accordingly, this secondary analysis shares the participant sample and QLQ-C30 data with the previous publication. The incremental contribution of the present study lies in the analysis of previously unreported psychological data. Specifically, the present analysis examines depressive symptoms using the PHQ-9, post-traumatic stress symptoms using the PCL-5, sociodemographic and clinical factors associated with these psychological outcomes, the relationship between depressive and post-traumatic stress symptoms, and their associations with HRQoL. The PHQ-9 and PCL-5 findings, along with the statistical analyses involving these psychological measures and their associations with HRQoL, have not been previously reported [25].

### 2.3. Sample Size

This study is a secondary analysis of a previously established cohort. During the original study period, the eligible BC population at the participating center consisted of 107 women, all of whom were invited and enrolled. For the original study, a population sample size estimation indicated that a minimum of 84 participants would be required to achieve a 95% confidence level with a 5% margin of error. No separate a priori sample size or power calculation was performed specifically for the present secondary psychological analyses; therefore, the available sample comprised the full cohort of 107 participants. Given the modest sample size relative to the number of variables included in the multivariable models, these analyses were considered exploratory. Hence, the multivariable findings were interpreted cautiously, particularly in view of the modest explanatory power of the models and for associations with *p*-values close to the conventional threshold for statistical significance.

### 2.4. Reduction of Potential Bias

Mitigation of both information and selection bias was in alignment with STROBE guidelines. Information bias was minimized by using validated Greek translations of the EORTC QLQ-C30 [26], PCL-5 [27,28], and PHQ-9 [29] questionnaires, along with uniform written instructions and consistent environmental conditions for all participants. Selection bias was addressed by recruiting eligible patients consecutively, based on clearly defined inclusion criteria. The consistent application of these criteria and the standardization of data collection methods ensured homogeneity among participants. Further, relevant demographic and clinical variables were included in multivariable linear regression analyses to account for potential confounding effects on PTSD and depression outcomes.

### 2.5. Data Collection and Verification

Missing data were assessed before statistical analysis, and none were identified because all questionnaires were completed under the supervision of the primary investigator (A.K.), who directly collected patients’ responses in hospital wards or outpatient clinics at Hippokration General Hospital. Completed questionnaires were immediately checked for completeness and accuracy, and the data were double-entered and cross-verified with the original forms, allowing analyses to be performed on complete datasets without the need for imputation.

### 2.6. General Questions Regarding Demographic and Other Personal Information

BC patients filled out a questionnaire that gathered demographic and clinical information, including age, smoking status, comorbidities, and details about surgery and chemotherapy (see Supplementary Materials Section S1).

### 2.7. Post-Traumatic Stress Checklist (PCL-5)

The PCL-5 is a 20-item self-report questionnaire designed to assess post-traumatic stress symptoms experienced during the past month. Each item is rated on a five-point Likert scale ranging from 0 (“Not at all”) to 4 (“Extremely”), yielding a total symptom severity score ranging from 0 to 80, with higher scores indicating greater post-traumatic stress symptom severity. For the DSM-5 symptom-cluster scoring algorithm, an item rated ≥2 (“Moderately”) was considered an endorsed symptom. The scoring rule requires at least one endorsed Criterion B symptom (intrusion; items 1–5), one Criterion C symptom (avoidance; items 6–7), two Criterion D symptoms (negative alterations in cognition and mood; items 8–14), and two Criterion E symptoms (alterations in arousal and reactivity; items 15–20) [30,31]. For the purposes of the present analyses, fulfillment of all four symptom-cluster requirements was used as a symptom-based screening classification for probable PTSD.

The PCL-5 is a self-report screening instrument and does not establish a clinical diagnosis of PTSD. DSM-5 Criterion A was not independently assessed, and no structured diagnostic interview, such as the CAPS-5, was conducted. Accordingly, the PCL-5 classification used in this study should be interpreted as indicating probable PTSD based on symptom criteria rather than clinically diagnosed PTSD. The Greek-language version of the PCL-5 used in the present study has previously undergone translation and psychometric evaluation in Greek women, supporting its use as a Greek-language measure of post-traumatic stress symptoms [27,28].

### 2.8. Patient Health Questionnaire-9 (PHQ-9)

The PHQ-9 is a self-administered questionnaire used to assess the severity of depressive symptoms over the past two weeks. It consists of 9 items, each corresponding to one of the DSM-IV criteria for depression. Each item is scored on a 4-point Likert scale ranging from 0 (“Not at all”) to 3 (“Nearly every day”), yielding a total score between 0 and 27. Higher scores indicate more severe depressive symptoms, with standard cutoff points commonly interpreted as follows: 5–9 (mild), 10–14 (moderate), 15–19 (moderately severe), and 20–27 (severe depression) [29,32,33].

### 2.9. EORTC QLG Core Questionnaire (EORTC QLQ-C30)

The EORTC QLQ-C30 is a 30-item questionnaire designed to assess the functional status and symptom burden that impact the QoL of individuals with cancer. It consists of three main components:

- The Global Health Status and QoL (GHS) scale.
- Functional scales evaluate five areas: physical functioning, role functioning, emotional functioning, cognitive functioning, and social functioning.
- Symptom scales that measure various issues, including fatigue, nausea and vomiting, pain, dyspnea, insomnia, appetite loss, constipation, diarrhea, and financial difficulties caused by physical condition or medical treatment.

In general, higher scores on the functional scales indicate better functioning, while higher scores on the global health status/QoL scale suggest better overall QoL. Conversely, higher symptom scale scores indicate greater severity of symptoms or related issues [34].

### 2.10. Ethical Approval

A detailed research protocol, along with relevant study documentation and the informed consent form (Section S2), was submitted to the Research Ethics Committee of the University of Western Macedonia, and ethical approval was granted on 31 March 2023 (Protocol No.: 178/2023). The submitted proposal included a comprehensive description of the study objectives, methodological framework, and procedures to ensure confidentiality and participant anonymity, in accordance with the General Data Protection Regulation and the Declaration of Helsinki. Approval was additionally obtained from the Hippokration Hospital Committee for Quality Assurance of Research and Continuing Education (No. 23794/22 May 2023).

### 2.11. Statistical Analysis

Statistical analyses were performed using IBM SPSS Statistics for Windows, Version 28.0 (IBM Corp., Armonk, NY, USA, 2022). Normality of continuous variables was assessed using the Kolmogorov–Smirnov test. Normally distributed quantitative variables are reported as means ± standard deviations (SD), while categorical variables are presented as frequencies (*n*) and percentages (%). Scale scores for the PCL-5, PHQ-9, and QLQ-C30 are reported as means ± SD, along with 95% confidence intervals (CI) and minimum and maximum values. No missing data were identified for these instruments. Internal consistency was evaluated using Cronbach’s alpha, with α ≥ 0.70 indicating acceptable reliability. Exploratory multiple linear regression analyses were performed to examine the adjusted associations of sociodemographic and clinical characteristics with depressive and post-traumatic stress symptom severity, using total PHQ-9 and total PCL-5 scores as dependent variables, respectively. Given the modest sample size relative to the number of variables included in the models, these analyses were considered exploratory. Therefore, associations with *p*-values close to the conventional significance threshold were interpreted cautiously. All independent variables were entered simultaneously into the models. These included age, employment status, educational level, marital status, place of residence, Charlson Comorbidity Index (CCI), surgical treatment, type of treatment received before chemotherapy, and time since BC diagnosis. Categorical variables with more than two categories were dummy-coded. The reference categories were as follows: employed for employment status, compulsory education for educational level, married for marital status, urban residence for place of residence, no mastectomy for surgical treatment, neither endocrine nor radiation therapy for treatment prior to chemotherapy, and >3 years for time since BC diagnosis. Multicollinearity was assessed using variance inflation factors (VIFs), and independence of residuals was evaluated using the Durbin–Watson statistic. Because all participants were receiving chemotherapy at the time of assessment, the adjusted association of chemotherapy exposure with PHQ-9 or PCL-5 scores could not be examined. Multivariate analysis of variance (general linear model) was conducted to assess the associations of probable PTSD status and depressive symptom severity with QoL outcomes, including functioning and symptom dimensions. Group comparisons for continuous outcomes (PHQ-9 and PCL-5 scores) were performed using one-way ANOVA for variables with three or more categories. An independent-samples *t*-test was used to compare depressive symptom severity between women meeting versus not meeting the PCL-5 DSM-5 symptom-cluster scoring rule for possible PTSD. Statistical significance was defined as *p* ≤ 0.05.

## 3. Results

### 3.1. Demographic and Clinical Data of Participants

A total of 107 participants voluntarily provided informed consent to participate in this study. All participants were adult women with a confirmed diagnosis of BC who were receiving chemotherapy at the time of study participation. Thus, all 107 women included in the final study sample were undergoing chemotherapy during the study period. Their demographic and personal characteristics are presented in Table 1.

### 3.2. PTSD Results

The descriptive statistics for the 20-item PCL-5 scale, which evaluates PTSD symptoms in BC patients based on DSM-5 criteria, are presented in Table 2 and Table 3. The findings indicate that the mean scores for most PCL-5 scale items fall within the range of 1 (slightly) to 2 (moderately), with some items exhibiting mean values marginally exceeding 2. Overall, these results suggest that post-traumatic stress symptomatology in this population tends to manifest at moderate levels.

**Figure 1:**
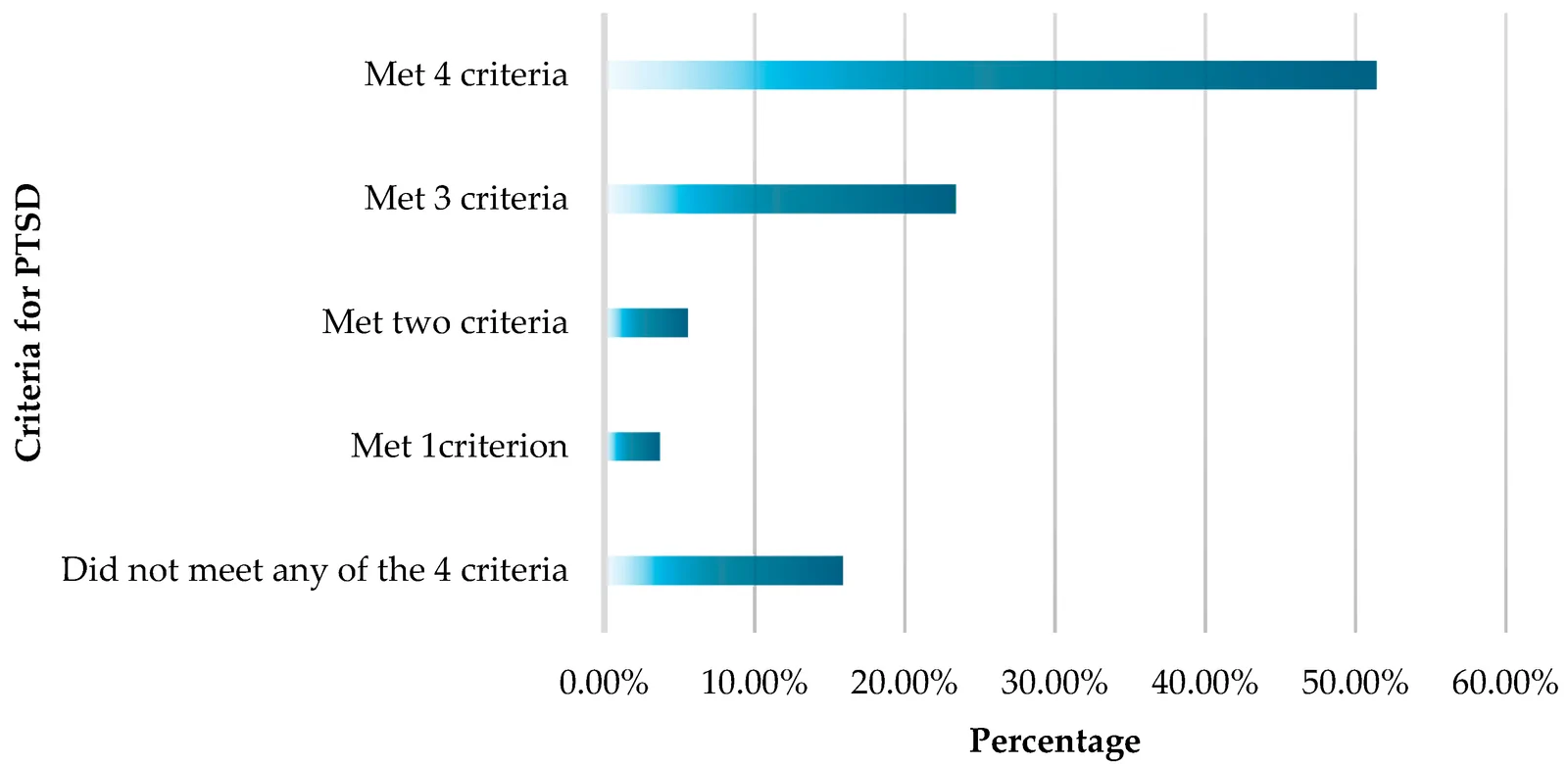
Results showing the number of criteria met by BC patients.

Additionally, most participants met the individual PCL-5 symptom-cluster criteria, while 51.4% met all four criteria used to classify probable PTSD (Table 4 and Figure 1). This classification should not be interpreted as a clinical diagnosis of PTSD.

### 3.3. Depression Results

Table 5 presents the results regarding the nine questions of the PHQ-9 scale used in the study, which evaluates depressive symptoms. These findings indicate that the average responses to most questions fall between 1 (some days) and 2 (more than half the days). Overall, it appears that the levels of depressive symptoms among women with BC vary. Moreover, the PHQ-9 demonstrated excellent internal consistency in this study, with Cronbach’s alpha coefficient of 0.903.

**Figure 2:**
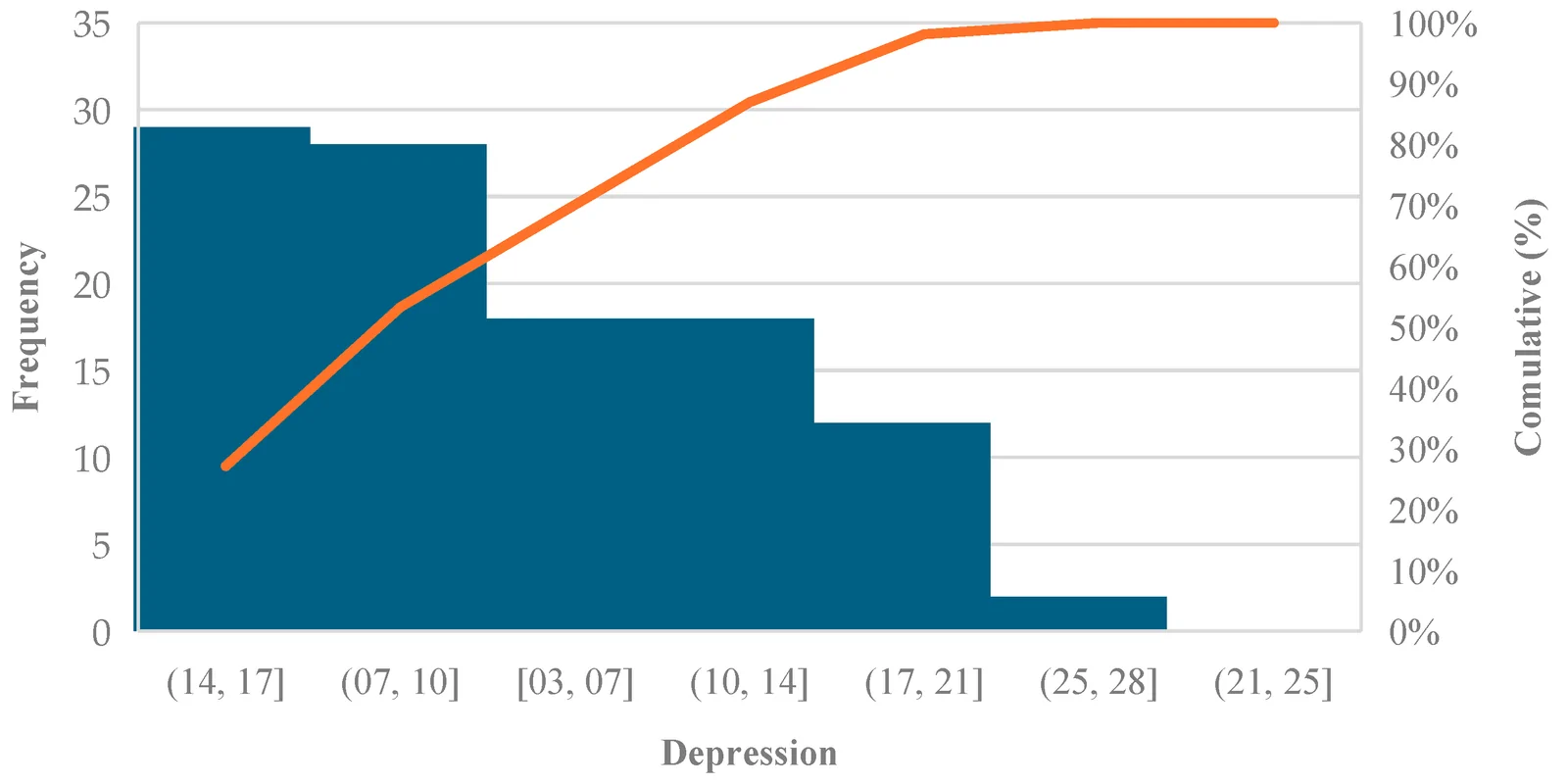
Distribution of the total depression score based on the PHQ-9 tool.

**Figure 3:**
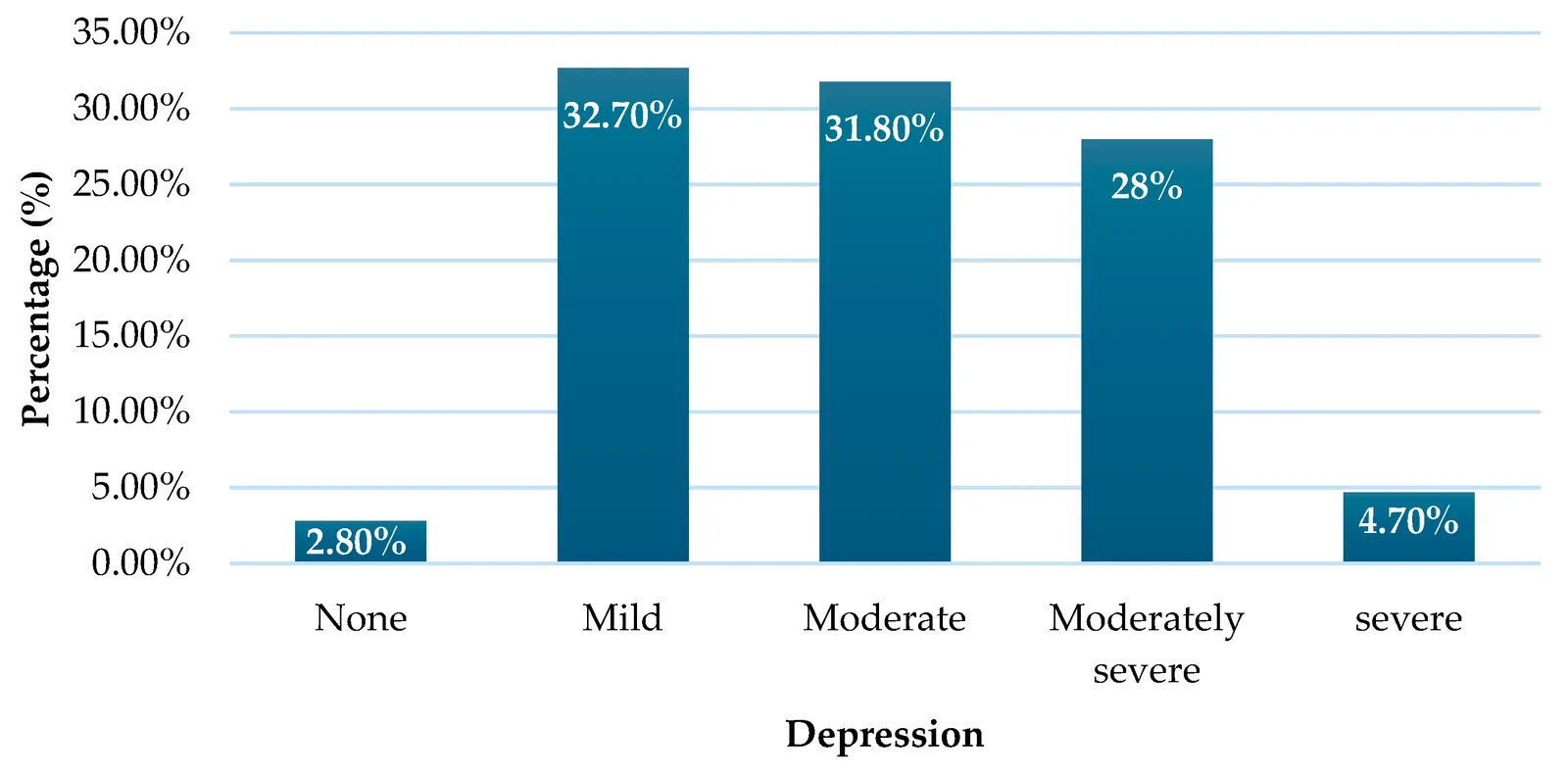
Results for the level of depression symptomatology in the women in the sample.

Moreover, the mean PHQ-9 score was 11.9 (SD = 4.9), with depressive symptom severity ranging from no symptoms to severe symptoms across the study population (Figure 2 and Figure 3).

### 3.4. HRQoL Results

HRQoL was moderate, with relatively higher physical and social functioning and lower emotional and cognitive functioning. Fatigue represented the most prominent symptom burden, while most other symptoms were reported at relatively low levels (Supplementary Table S1). Overall, the QLQ-C30 scale showed excellent internal consistency in this study, with Cronbach’s alpha coefficient of 0.919 [25].

### 3.5. Sociodemographic and Clinical Factors Associated with Depressive and Post-Traumatic Stress Symptom Severity

**Figure 4:**
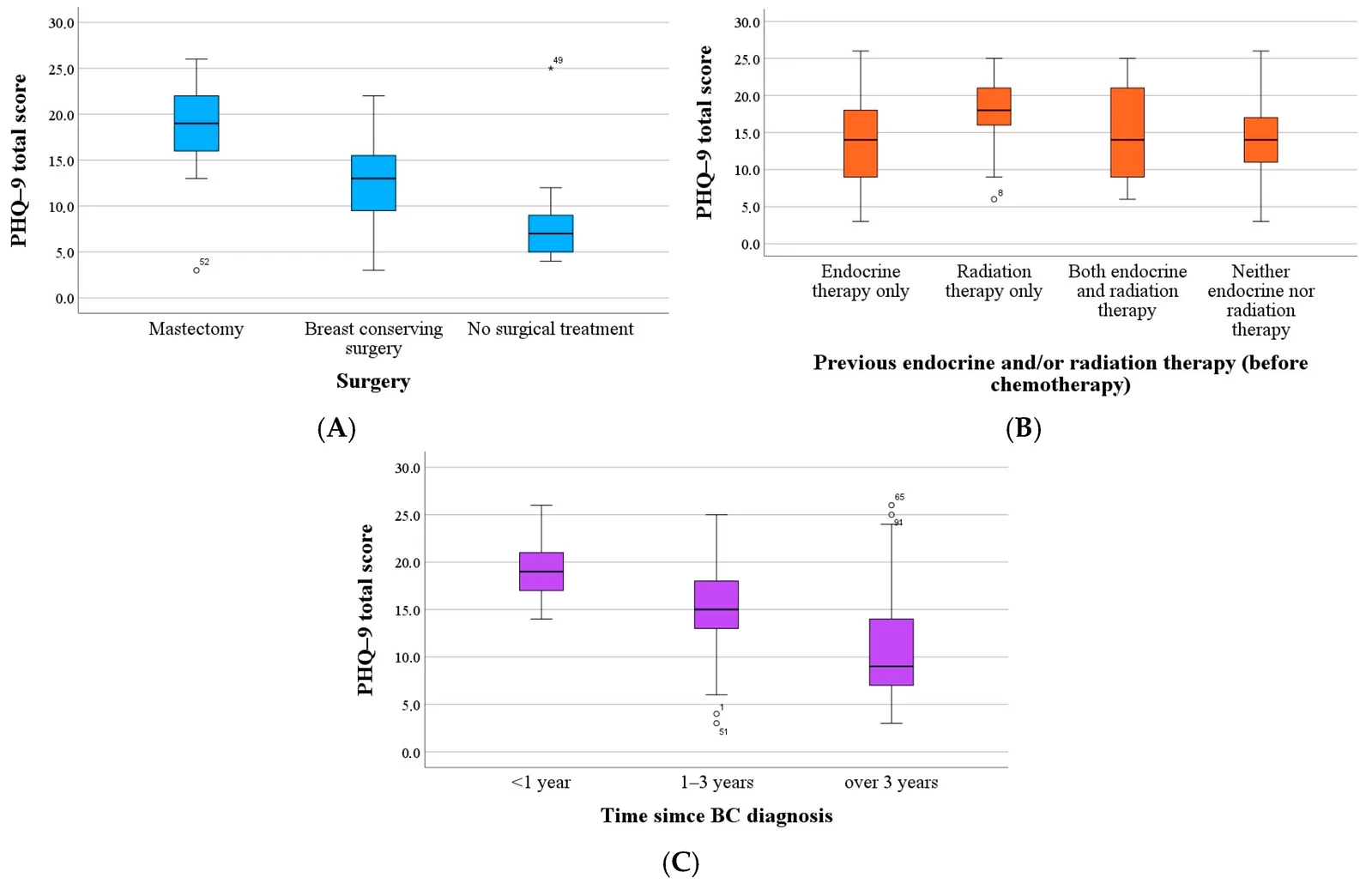
(**A**–**C**). Scatterplots depicting the relationship between different clinical data and PHQ-9 reported scores. (**A**) presents PHQ-9 scores according to type of surgical treatment (mastectomy, breast-conserving surgery, and no surgical treatment). (**B**) shows PHQ-9 scores based on previous endocrine and/or radiation therapy before chemotherapy (endocrine therapy only, radiation therapy only, both endocrine and radiation therapy, or neither therapy). (**C**) illustrates PHQ-9 scores according to time since BC diagnosis (<1 year, 1–3 years, and >3 years). Higher PHQ-9 scores indicate greater severity of depressive symptoms. ○ indicates outliers and * indicates extreme outliers. Statistical significance was assessed using one-way ANOVA, with a significance threshold set at *p* ≤ 0.05.

An exploratory multiple linear regression analysis was performed to examine sociodemographic and clinical factors independently associated with depressive symptom severity. The overall model was statistically significant (R^2^ = 0.242; adjusted R^2^ = 0.106; *p* = 0.047), although its explanatory power was modest. After adjustment for the other variables included in the model, mastectomy was associated with higher PHQ-9 scores compared with no mastectomy (B = 2.416, β = 0.245, 95% CI: 0.319–4.513, *p* = 0.024). Women assessed 1–3 years after BC diagnosis also had higher PHQ-9 scores compared with those assessed more than 3 years after diagnosis (B = 2.257, β = 0.228, 95% CI: 0.020–4.495, *p* = 0.048). In contrast, endocrine therapy alone was associated with lower PHQ-9 scores than receiving neither endocrine nor radiation therapy (B = −2.413, β = −0.217, 95% CI: −4.771 to −0.055, *p* = 0.045). The remaining variables in the model showed no statistically significant independent associations (Supplementary Table S2). Unadjusted comparisons according to surgery, previous treatment, and time since diagnosis are presented in Figure 4.

**Figure 5:**
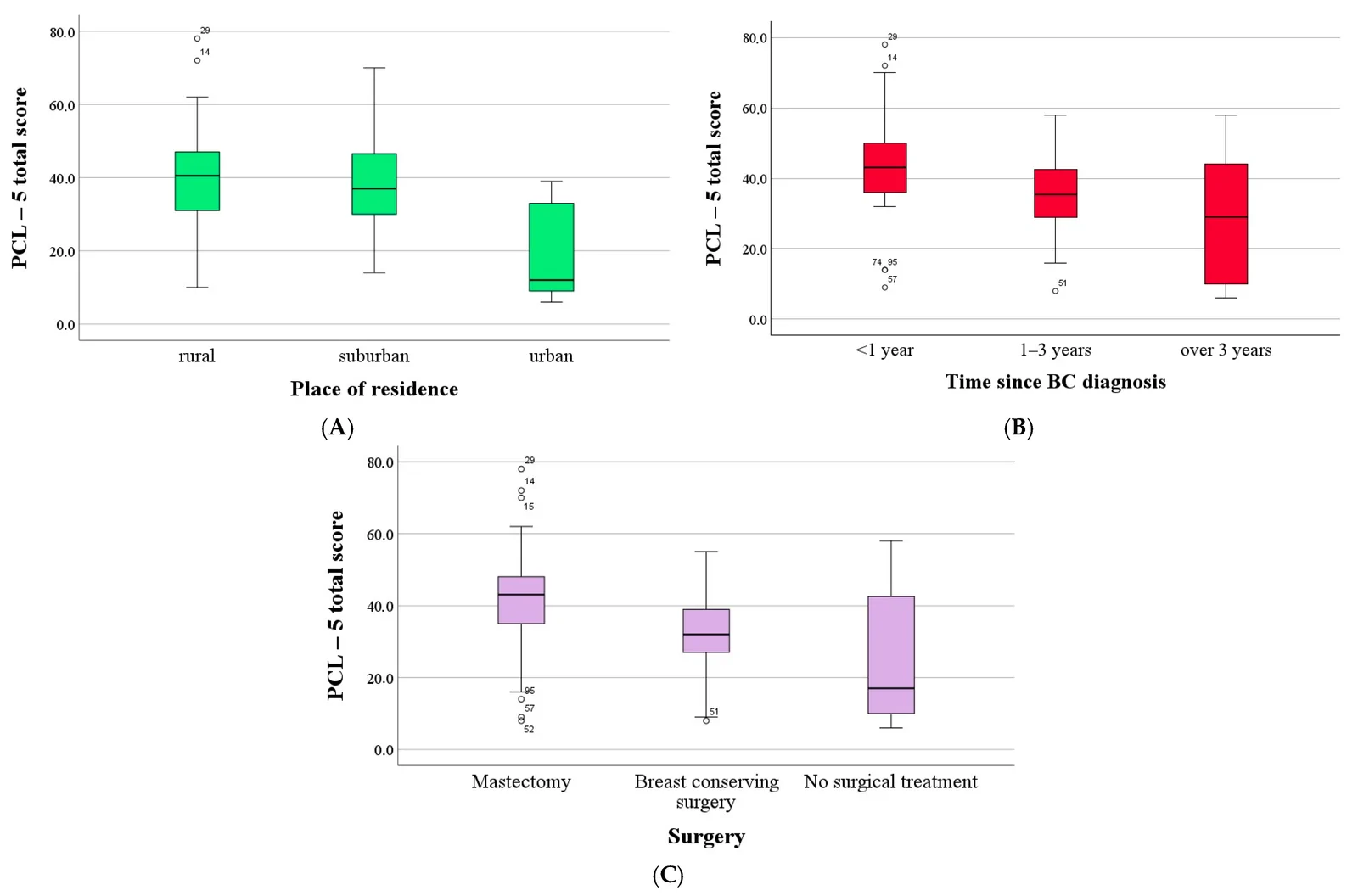
(**A**–**C**). Scatterplots depicting the relationship between different demographic/clinical data and PCL-5 reported scores. (**A**) presents PCL-5 scores according to place of residence (urban, suburban, rural). (**B**) shows PCL-5 scores based on time since BC diagnosis (<1 year, 1–3 years, and >3 years). (**C**) illustrates PCL-5 scores according to the type of surgical treatment (mastectomy, breast-conserving surgery, and no surgical treatment). Higher PCL-5 scores indicate greater severity of post-traumatic stress symptoms. ○ indicates outliers. Statistical significance was assessed using independent *t*-tests or one-way ANOVA, as appropriate, with *p* ≤ 0.05.

An exploratory multiple linear regression analysis was also performed to identify factors associated with post-traumatic stress symptom severity. In total, the model was statistically significant (R^2^ = 0.247, adjusted R^2^ = 0.112, *p* = 0.039). Results showed that rural residence was linked to higher PCL-5 scores than urban residence (B = 12.758, *p* = 0.007). Women who had a mastectomy exhibited higher scores than those who did not (B = 7.134, *p* = 0.038). Additionally, women assessed 1–3 years after breast cancer diagnosis had higher PCL-5 scores than those assessed more than 3 years post-diagnosis (B = 6.995, *p* = 0.048). Finally, the model found no significant associations for the other variables (Supplementary Table S3). Corresponding unadjusted comparisons of PCL-5 scores according to residence, time since diagnosis, and surgery are presented in Figure 5.

### 3.6. Multivariate Analysis Between PTSD, Depression and HRQoL

PTSD status was significantly associated with poorer HRQoL across all functional dimensions of the EORTC QLQ-C30 and with greater fatigue, nausea/vomiting, pain, dyspnea, insomnia, appetite loss, and financial difficulties. No significant associations were observed for constipation or diarrhea (Table 6 and Supplementary Table S4).

Likewise, greater depressive symptom severity was significantly associated with poorer HRQoL across all functional dimensions and with a greater burden of most symptoms assessed by the EORTC QLQ-C30. Constipation and diarrhea were not significantly associated with depressive symptom severity (Table 7 and Supplementary Table S5).

### 3.7. Correlation Between PTSD and Depression Levels

**Figure 6:**
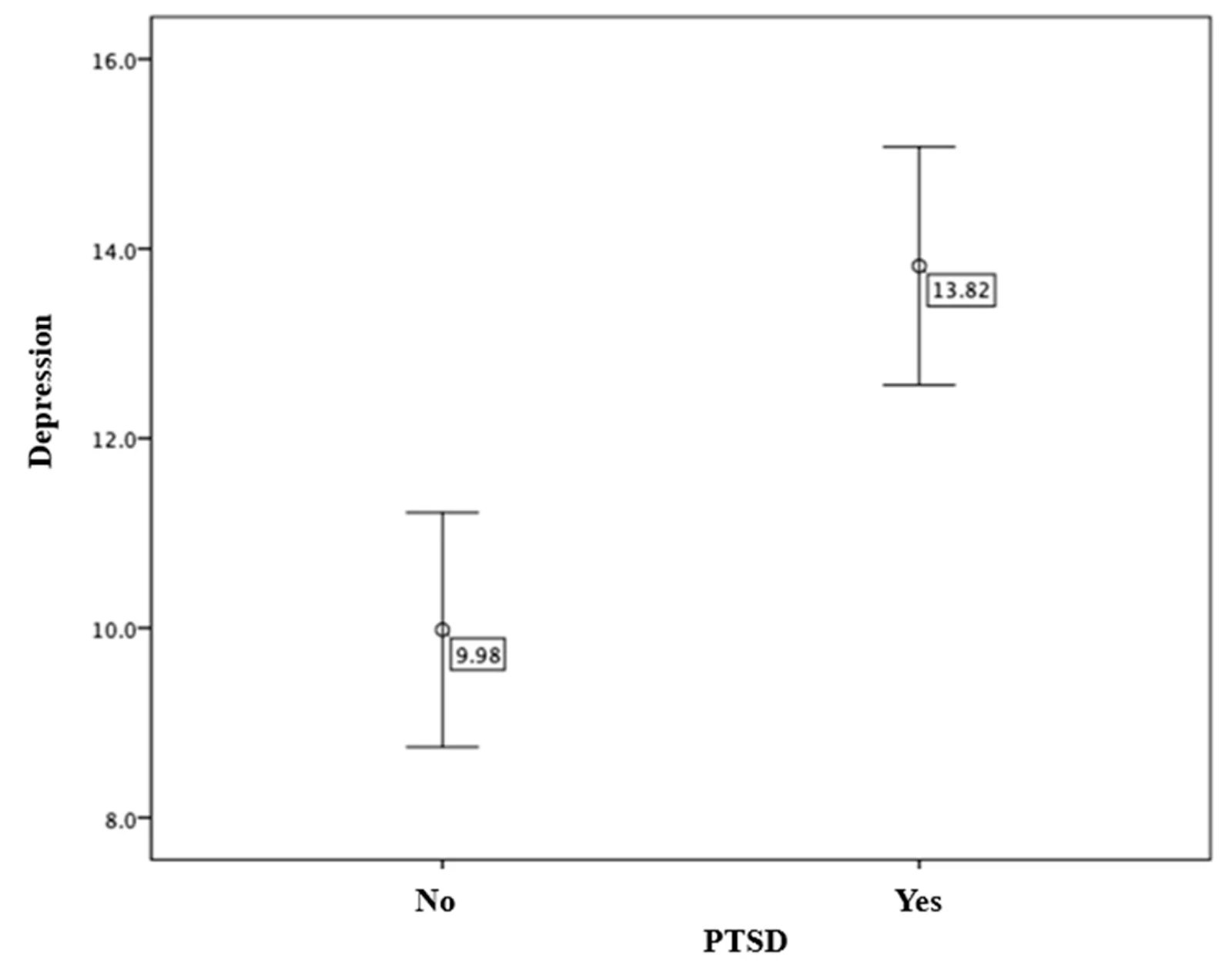
Comparison of PHQ-9 scores according to PCL-5 probable PTSD classification. The bars illustrate the mean total depression score, along with the 95% confidence interval, for women with PTSD compared to those without PTSD.

**Figure 7:**
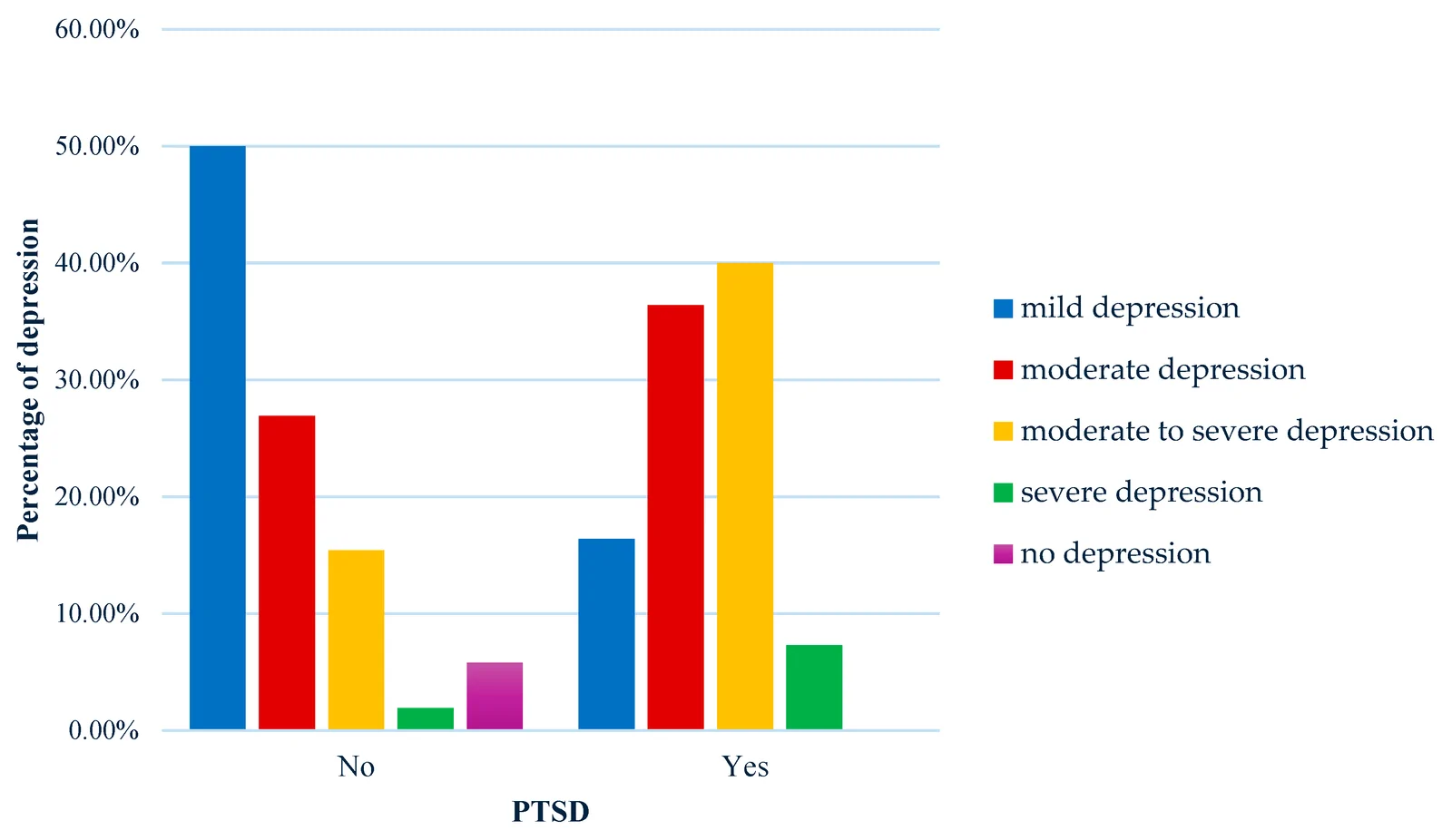
Severity of depressive symptoms according to PCL-5 probable PTSD classification.

Women meeting the criteria for probable PTSD had significantly higher PHQ-9 scores than those without probable PTSD (Figure 6). Depression severity was also significantly associated with probable PTSD status (χ^2^ test, *p* < 0.001; Figure 7).

## 4. Discussion

The diagnosis of BC, recognized as a major traumatic event necessary for the development of PTSD, contributes significantly, alongside depression, to the deterioration of overall QoL in BC patients. This cross-sectional secondary analysis of a previously described cohort of 107 Greek women with BC provides additional psychological data not examined in the previous publication. Particularly, we evaluated depressive and post-traumatic stress symptoms, their interrelationship, and their associations with HRQoL, together with exploratory associations between selected sociodemographic and clinical characteristics and psychological symptom severity. Interpret the findings in the context of the relatively modest sample size, the secondary nature of the analysis, and the cross-sectional design.

Our study, based on PHQ-9 scores, found that most BC patients had mean values below the clinical threshold for major depressive disorder. Nevertheless, depressive symptoms were present across a broad severity spectrum, from mild to severe, with only 2.8% of participants reporting no symptoms, indicating a high prevalence of psychological distress. In the adjusted analysis, mastectomy and an interval of 1–3 years since BC diagnosis were independently associated with higher PHQ-9 scores compared with no mastectomy and >3 years since diagnosis, respectively. In contrast, previous endocrine therapy alone was associated with lower PHQ-9 scores compared with receipt of neither endocrine nor radiation therapy. These findings should be interpreted cautiously, given the multivariable model’s modest explanatory power and the study’s cross-sectional design. However, the psychological distress observed in the present cohort cannot be attributed specifically to chemotherapy, since all participants were receiving chemotherapy at the time of assessment. Therefore, the independent contribution of chemotherapy could not be distinguished from the broader psychological burden associated with cancer diagnosis and treatment, including repeated hospital attendance, treatment-related symptoms, and other psychosocial stressors. A recent systematic review [35] reported substantial regional variation in depression prevalence among BC patients and survivors. Patient prevalence ranged from 6.6% in Italy and 25.5% in Portugal to approximately 35% in Syria and Palestine, suggesting higher rates in Middle Eastern contexts compared to Southern Europe. In contrast, survivors generally reported lower rates of depression, with Germany having the highest rate at 34.9%, while Greece had a much lower rate of 18.2%. Upon closer examination, various clinical and demographic factors showed significant associations with depression levels. It turned out that younger individuals, those who had been employed before, people with lower educational backgrounds, and those who underwent chemotherapy or mastectomy were more likely to experience higher levels of depression [35]. In a comparable study conducted in Greece [3], 38.2% of BC patients showed depressive symptoms, with an average PHQ-2 score of 2.05. Univariate analysis identified age, marital status, education, and cancer stage as related to depression, while multivariate analysis revealed that rural residence, religious affiliation, and symptom burden were independent predictors of depressive severity [3]. Similarly, another Greek cohort study reported a mean PHQ-2 score of 2.2 (SD = 1.8) among cancer patients, with 54.5% indicating a depressed mood [24]. Research also indicates that the prevalence of depression among Serbian BC patients ranges from 25% to 27%. Symptom severity ranges from moderate to severe and is significantly associated with sociodemographic factors such as age, occupation, and educational level [36]. Additionally, over half of the Palestinian women who were aware of their BC stage and type reported significantly elevated levels of depression [37]. This suggests that depressive symptoms in BC patients are likely multifactorial and may be influenced by disease-related, treatment-related, and psychosocial factors rather than by chemotherapy alone [37].

Furthermore, our study demonstrated that greater depressive symptom severity was significantly associated with poorer QoL among BC patients. Multivariate analysis revealed that nearly all subscales of the EORTC QLQ-C30, both functional and symptom-related, were significantly associated with total PHQ-9 scores, with the exception of the constipation and diarrhea subscales. These findings are consistent with previous research, which underscores depression as a critical determinant of QoL in BC patients. Depression can significantly impair patients’ psychological resilience and motivation to engage in treatment, while also diminishing the overall QoL in survivors, even after successful completion of therapy [35,37]. Evidence consistently indicates a strong relationship between depression and reduced QoL in BC patients. In a Greek study, anxiety and depressive symptoms were strongly and negatively associated with overall QoL and nearly all EORTC QLQ-C30 domains, although the single-hospital convenience sample limits generalizability [23]. Similar findings from larger studies show that depressed BC patients report significantly poorer QoL across multiple domains during active treatment [15]. Likewise, depressive symptoms have been inversely associated with HRQoL indicators such as functional status, financial well-being, and global health, while correlating with greater symptom burden [38]. Among BC survivors, depressive symptoms, reported in about 17.1%, are linked to persistently lower QoL across domains, although some functional differences may diminish after adjusting for factors such as physical activity [39].

Regarding the presence of probable PTSD, in our sample, 51.4% of participants met all four PCL-5 DSM-5 symptom-cluster requirements. This relatively high proportion should be interpreted as a symptom-based screening classification rather than the prevalence of clinically diagnosed PTSD, since Criterion A was not independently assessed and no structured diagnostic interview was conducted. In the adjusted analysis, rural residence, mastectomy, and an interval of 1–3 years since BC diagnosis were independently associated with higher PCL-5 scores compared with urban residence, no mastectomy, and >3 years since diagnosis, respectively. Notably, an interval of less than 1 year since diagnosis was not independently associated with PCL-5 scores after adjustment for the other sociodemographic and clinical variables included in the model. A meta-analysis of 38 studies reported that approximately 10% of women develop PTSD following a BC diagnosis [40]. However, prevalence estimates vary considerably across studies, ranging from 0% to 32.3%, likely due to differences in methodological approaches and inclusion criteria [41]. This wide variation may reflect not only differences in how the BC diagnosis is experienced as a Criterion A traumatic event but also the influence of additional psychosocial and clinical risk factors. Notably, individuals diagnosed with cancer have a 1.66-fold increased lifetime risk of developing PTSD compared to those without a cancer history [40]. This elevated risk underscores the significant psychological burden associated with cancer diagnosis and treatment, even when compared to other traumatic experiences. In a longitudinal study that aimed to assess PTSD symptom prevalence in female BC patients before surgery and one year postoperatively, researchers found that PTSD symptoms significantly declined over the year. Initially, higher symptom levels were associated with employment status, a history of emotional trauma, and dissatisfaction with information delivery. After one-year, increased symptoms were linked to breast-conserving surgery, being in a partnership, higher education, unemployment, and exposure to severe trauma in the preceding year [42]. A prospective study reported that PTSD prevalence among newly diagnosed female BC patients decreased over time, from 46% at baseline to 31% at 3 months and 22% at 6 months. At the six-month follow-up, PTSD was significantly associated with factors such as religiosity, treatment accessibility, illness perception, receipt of chemotherapy, and QoL [43].

The relatively high proportion of women who had undergone mastectomy (60.7%) should also be considered when interpreting the psychological findings. Although contemporary breast cancer management increasingly favors surgical de-escalation and breast-conserving approaches in appropriately selected patients, detailed information on the clinical factors determining the type of surgery in the present cohort was not available [44]. In the adjusted analyses, mastectomy remained independently associated with both higher PHQ-9 and higher PCL-5 scores compared with no mastectomy, after accounting for the measured sociodemographic and clinical characteristics. Yet, given the cross-sectional design, these findings indicate associations rather than causal effects of mastectomy on psychological outcomes. Moreover, information on tumor stage and other factors influencing the choice of surgical procedure was not available, and residual confounding cannot therefore be excluded.

These findings are consistent with our results, where both depression and PTSD were associated with poorer HRQoL, as measured by the EORTC QLQ-C30, highlighting the substantial psychological burden of BC. At the same time, patients overall reported moderate QoL levels. Previous studies show that PTSD is negatively correlated with HRQoL, with higher symptom severity linked to poorer QoL outcomes [45]. Likewise, significant inverse associations between QoL domains and PTSD severity have been reported, with many patients experiencing poor global QoL and physical functioning, alongside symptoms such as insomnia, fatigue, and appetite loss criteria [46]. Regarding the association between QoL and depression, in the Indian area, evidence indicates that 21.5% of patients showed symptoms of depression, with 22% of these cases classified as moderately severe to severe [15], highlighting its detrimental impact on both functional status and psychosocial well-being.

Moreover, our findings indicate that women with BC who met the PCL-5 symptom-cluster criteria for probable PTSD reported significantly higher levels of depressive symptoms compared with those without probable PTSD symptomatology. This observation aligns with previous research showing a close relationship between PTSD and depression among cancer patients, despite PTSD being less frequently examined in this population [42]. Furthermore, evidence suggests that depression is associated with increased PTSD symptomatology in BC patients and this correlation also extends to the caregivers of these patients, underscoring the broader psychosocial implications of these comorbid conditions [47].

To sum up, this study has both strengths and limitations. It demonstrated a significant association between HRQoL, depression, and PTSD symptoms among Greek BC patients. It identified mastectomy, place of residence, and time since BC diagnosis as factors independently associated with psychological symptom severity in the adjusted analyses. However, these findings should not be interpreted as causal. The relatively small sample sizes within treatment subgroups may have reduced the statistical power to detect subtle but clinically relevant differences, thereby limiting the robustness of between-group comparisons. First of all, a significant limitation is the secondary-analysis design, which restricted the present analyses to variables collected in the original study protocol. Thus, several potentially relevant clinical and psychosocial factors were unavailable, including family and social support, employment continuity, breast reconstruction, coping strategies, resilience, previous psychiatric history, recurrence status, and treatment intensity. Their absence limits a more comprehensive assessment of psychological adaptation and may have contributed to residual confounding. Secondly, the multivariable models demonstrated limited explanatory power, and several associations had *p*-values near the conventional significance threshold; thus, these findings should be considered exploratory. Moreover, the lack of data on cancer staging limits the ability to interpret variations in QoL outcomes across different disease trajectories fully. Because all participants received chemotherapy, the independent psychological contribution of chemotherapy could not be estimated without a non-chemotherapy comparison group. We also did not assess treatment-context factors such as repeated hospital attendance, cumulative treatment burden, exposure to other patients’ illness experiences, and perceived treatment-related stress. They may have contributed to the observed psychological burden. Although mastectomy remained associated with PHQ-9 and PCL-5 scores after adjustment for measured sociodemographic and clinical variables, the cross-sectional design, lack of detailed tumor-stage information, and missing data on other determinants of surgical selection preclude causal inference and do not rule out residual confounding. Last but not least, several potentially relevant psychosocial and clinical variables were not assessed, including family and social support, employment continuity following diagnosis, breast reconstruction, coping strategies, resilience, previous psychiatric history, recurrence status, and treatment intensity. The absence of these factors limits our ability to characterize psychological adaptation more comprehensively and may have resulted in residual confounding of the observed associations. Lastly, PTSD status was based on a self-reported PCL-5 symptom-cluster classification rather than a structured clinical diagnostic interview. In addition, DSM-5 Criterion A was not independently assessed; therefore, the proportion meeting the PCL-5 symptom-cluster rule should not be interpreted as the prevalence of clinically diagnosed PTSD.

In the future, studies should include larger, multicenter, and socioeconomically diverse samples to enhance the generalizability of the findings. Longitudinal assessments conducted before, during, and after treatment could clarify changes in emotional morbidity and HRQoL and identify periods of greatest vulnerability. Future research should also incorporate structured clinical interviews and assess a broader range of clinical and psychosocial factors, including cancer stage, recurrence status, treatment intensity, previous psychiatric history, breast reconstruction, employment continuity, family and social support, coping strategies, and resilience. Inclusion of these variables may provide a more comprehensive understanding of psychological adaptation and help identify women with greater supportive care needs. Randomized controlled trials are warranted to evaluate interventions such as cognitive–behavioral therapy, counseling, peer-support programs, and digitally delivered psycho-oncology services, particularly among women undergoing mastectomy, women during the early years following BC diagnosis, and those living in rural or geographically underserved areas.

## 5. Conclusions

This cross-sectional secondary analysis provides additional evidence on depressive and post-traumatic stress symptoms and their associations with HRQoL in a previously described cohort of Greek women with BC receiving chemotherapy. Exploratory multivariable analyses suggested associations between selected sociodemographic and clinical characteristics and psychological symptom severity; however, these findings require confirmation in larger prospective studies. The findings support the relevance of psychological assessment as part of comprehensive BC care. Greater emotional distress was associated with poorer global health, reduced physical, role, emotional, cognitive, and social functioning, and a higher symptom burden. Mastectomy and an interval of 1–3 years since diagnosis were linked to more pronounced distress, whereas rural residence was associated with elevated post-traumatic stress scores. Women meeting the criteria for probable PTSD also reported more severe depressive symptoms, highlighting the close relationship between these conditions. Finally, the multivariable models show limited explanatory power, and several estimates have only marginal statistical significance. Therefore, interpret these subgroup associations with caution and treat them as exploratory.

These findings support the relevance of standardized mental health assessment within comprehensive oncology care. Screening should be accompanied by clear referral pathways to psycho-oncology and multidisciplinary services. Larger prospective studies are needed to determine whether particular clinical or sociodemographic subgroups have greater psychological supportive care needs.

## Figures and Tables

**Table 1:** Demographic and clinical data in BC patients.

BC Patients (*n* = 107)	
Demographic variable	
Age	58.1 ± 11.9
Marital status	
Single	29 (27.1%)
Married	49 (45.8%)
Divorced	16 (15.0%)
Widow	13 (12.1%)
Educational level	
Compulsory (primary/secondary) education	55 (51.4%)
Higher (Bachelor’s) degree	39 (36.4%)
Master’s or PhD	13 (12.1%)
Employment status	
Employed	22 (20.6%)
Unemployed	85 (79.4%)
Place of residence	
Urban area	65 (60.7%)
Suburban area	28 (26.2%)
Rural area	14 (13.1%)
Smoking	
Yes	19 (17.7%)
No	88 (82.24%)
Clinical	
Time since BC diagnosis	
<1 year	24 (22.4%)
≥1 year and <3 years	46 (42.9%)
≥3 years	37 (34.5%)
Previous endocrine and/or radiation therapy (before chemotherapy)	
Endocrine therapy only	36 (33.6%)
Radiation therapy only	25 (23.4%)
Both endocrine and radiation therapy	30 (28.0%)
Neither endocrine nor radiation therapy	16 (15.0%)
Surgery	
Mastectomy	65 (60.7%)
Breast conserving surgery	39 (36.4%)
No surgical treatment	3 (2.8%)
Presence of Comorbidities	
Yes	46 (42.9%)
No	61 (57.0%
Specific Comorbidities	
Hypertension	38 (35.5%)
Thyroid disease	15 (14.0%)
Type II Diabetes mellitus	12 (11.2%)
Chronic lung disease	4 (3.7%)
Dyslipidemia	35 (32.7%)
Heart failure	3 (2.8%)
Coronary artery disease	6 (5.6%)
Anemia	9 (8.4%)
Osteoporosis	4 (3.7%)
Gastrointestinal disorders	6 (5.6%)
Other	2 (1.9%)
CCI	7.3 ± 0.9

Note: BC: Breast Cancer; CCI: Charlson Comorbidity Index. Data are expressed as mean ± standard deviation and as frequencies (*n*) and percentages (%).

**Table 2:** Descriptive analysis results for the 20 questions of the PCL-5 scale.

	M	SD
Criteria B		
Repeated, disturbing, and unwanted memories of the stressful experience?	2.0	0.9
Repeated, disturbing dreams of the stressful experience?	1.5	1.1
Suddenly feeling or acting as if the stressful experience were actually happening again (as if you were actually back there reliving it)?	1.9	1.0
Feeling very upset when something reminds you of the stressful experience?	2.0	1.0
Having strong physical reactions when something reminds you of stressful experiences (for example, heart pounding, trouble breathing, sweating)?	1.8	1.1
Criteria C		
Avoiding memories, thoughts, or feelings related to the stressful experience?	2.3	1.0
Avoiding external reminders of stressful experiences (for example, people, places, conversations, activities, objects, or situations)?	2.3	1.0
Criteria D		
Trouble remembering important parts of the stressful experience?	2.2	1.0
Having strong negative beliefs about yourself, other people, or the world (for example, having thoughts such as: I am bad, there is something seriously wrong with me, no one can be trusted, the world is completely dangerous)?	1.3	1.0
Blaming yourself or someone else for the stressful experience or what happened after it?	1.4	1.1
Having strong negative feelings such as fear, horror, anger, guilt, or shame?	1.4	1.1
Loss of interest in activities that you used to enjoy?	2.3	1.1
Feeling distant or cut off from other people?	2.3	1.2
Trouble experiencing positive feelings (for example, being unable to feel happiness or have loving feelings for people close to you)?	2.2	1.2
Criteria E		
Irritable behavior, angry outbursts, or acting aggressively?	2.0	1.1
Taking too many risks or doing things that could cause you harm?	0.5	0.9
Being “superalert” or watchful or on guard?	1.5	1.2
Feeling jumpy or easily startled?	1.3	1.3
Having difficulty concentrating?	1.7	1.3
Trouble falling or staying asleep?	1.0	1.2

Note: M: mean; SD: standard deviation.

**Table 3:** Aggregate results for the four subdimensions of the PCL-5 scale.

	M	SD	Min	Max	Reliability via Cronbach’s Alpha
Criteria B	3.3	2.2	0.0	5.0	0.805
Criteria C	1.6	0.8	0.0	2.0	0.813
Criteria D	4.3	2.5	0.0	7.0	0.806
Criteria E	2.4	2.0	0.0	6.0	0.814

Note: M: mean; SD: standard deviation; Min: minimum value; Max: maximum value.

**Table 4:** Proportion of participants meeting the PCL-5 DSM-5 symptom-cluster criteria.

	No		Yes	
	*n*	%	*n*	%
Criteria B	29	27.1%	78	72.9%
Criteria C	23	21.5%	84	78.5%
Criteria D	22	20.6%	85	79.4%
Criteria E	43	40.2%	64	59.8%

Note: Data are expressed as frequencies (*n*) and percentages (%).

**Table 5:** Descriptive analysis results for each question of the PHQ-9 scale.

	M	SD
Little interest or pleasure in doing things	1.9	0.8
Feeling down, depressed, or hopeless	1.9	0.8
Trouble falling or staying asleep, or sleeping too much	1.4	0.8
Feeling tired or having little energy	2.3	0.7
Poor appetite or overeating	0.9	0.9
Feeling bad about yourself—or that you are a failure or have let yourself or your family down	1.4	1.1
Trouble concentrating on things, such as reading the newspaper or watching television	1.1	0.9
Moving or speaking so slowly that other people could have noticed? Or the opposite—being so fidgety or restless that you have been moving around a lot more than usual	0.6	0.8
Thoughts that you would be better off dead or of hurting yourself in some way	0.5	0.7
Total Score of PHQ-9	11.9	4.9

Note: M: mean; SD: standard deviation.

**Table 6:** Multivariate analysis of variance with dependent variables the dimensions of QoL and independent the presence of PTSD, as calculated with PCL-5.

Dependent Variable	Type III Sum of Squares	df	Mean Square	F	*p*	Partial Eta Squared
Global health status/QoL	8173.3	1	8173.3	27.517	0.000 *	0.211
Physical function	6843.1	1	6843.1	27.301	0.000 *	0.210
Role function	13,890.2	1	13,890.2	26.006	0.000 *	0.202
Emotional function	6891.9	1	6891.9	9.129	0.003 *	0.081
Cognitive function	10,308.2	1	10,308.2	14.789	0.000 *	0.126
Social function	8156.5	1	8156.5	9.198	0.003 *	0.082
Fatigue	19,225.6	1	19,225.6	36.256	0.000 *	0.260
Nausea and vomiting	1731.3	1	1731.3	7.524	0.007 *	0.068
Pain	8178.9	1	8178.9	13.554	0.000 *	0.116
Dyspnoea	5283.1	1	5283.1	10.015	0.002 *	0.089
Insomnia	9536.8	1	9536.8	12.385	0.001 *	0.107
Appetite loss	2919.6	1	2919.6	7.876	0.006 *	0.071
Constipation	2247.0	1	2247.0	3.376	0.069	0.032
Diarrhea	38.4	1	38.4	0.395	0.531	0.004
Financial difficulties caused by physical condition or medical treatment	3242.8	1	3242.8	4.902	0.029 *	0.045

Note: * Significant at *p* < 0.05.

**Table 7:** Multivariate analysis of variance with dependent variables the dimensions of QoL and independent the presence of depression, as calculated with PHQ-9.

Dependent Variable	Type III Sum of Squares	df	Mean Square	F	*p*	Partial Eta Squared
Global health status/QoL	10,774.3	2	5387.1	19.630	0.000 *	0.278
Physical function	9402.5	2	4701.3	20.618	0.000 *	0.288
Role function	21,267.3	2	10,633.6	22.768	0.000 *	0.309
Emotional function	10,134.8	2	5067.4	6.937	0.001 *	0.120
Cognitive function	9337.7	2	4668.8	6.545	0.002 *	0.114
Social function	22,186.3	2	11,093.2	14.636	0.000 *	0.223
Fatigue	17,256.4	2	8628.2	15.553	0.000 *	0.234
Nausea and vomiting	2206.7	2	1103.4	4.845	0.010 *	0.087
Pain	16,246.9	2	8123.4	15.320	0.000 *	0.231
Dyspnoea	16,313.1	2	8156.6	19.211	0.000 *	0.274
Insomnia	16,211.7	2	8105.8	11.383	0.000 *	0.182
Appetite loss	5633.0	2	2816.5	8.100	0.001 *	0.137
Constipation	1502.8	2	751.4	1.106	0.335	0.021
Diarrhea	158.6	2	79.3	0.818	0.444	0.016
Financial difficulties caused by physical condition or medical treatment	9175.1	2	4587.5	7.522	0.001 *	0.129

Note: * Significant at *p* < 0.05.

## Data Availability

The original contributions presented in this study are included in the article/Supplementary Materials. Further inquiries can be directed to the corresponding authors.

## Supplementary Materials

The following supporting information can be downloaded at https://www.mdpi.com/article/10.3390/medsci14060625/s1: Section S1: Demographic and Clinical Information Questionnaire (English Version); Section S2: Signed Informed Consent Form (English version); Table S1. Reliability and summary results for the subdimensions of the QLQ-C30 scale, evaluating HRQoL; Table S2: Multiple regression analysis with total PHQ-9 score, evaluating depression, as a dependent variable; Table S3: Multiple regression analysis with total PCL-5 score, evaluating PTSD, as a dependent variable; Table S4: Associations between probable PTSD status and QoL dimensions (functioning and symptoms); Table S5: Associations between depressive symptom severity and QoL dimensions (functioning and symptoms).
