# Supplementary material for: Depressive and Post-Traumatic Stress Symptoms and Their Associations with Health-Related Quality of Life in Women with Breast Cancer: A Cross-Sectional Secondary Analysis
Source: Med Sci (Basel). 2026 Sep 30;14(6):625. doi: 10.3390/medsci14060625 (PMC13706776; doi:10.3390/medsci14060625)
Supplement: Supplementary file 1 [file medsci-14-00625-s001.zip › medsci-4575657-supplementary.pdf]

Section S1

Demographic and Clinical Information Questionnaire (English Version)

Please complete the following questionnaire. Tick or fill in the appropriate answer. All responses will remain confidential and will be used only for research purposes.

Demographic Information

Age (years): ____________________

Marital status:☐ Single ☐ Married ☐ Divorced ☐ Widowed

Educational level:☐ Compulsory education ☐Bachelor's degree ☐ Master's/PhD

Place of residence:☐ Urban ☐ Suburban ☐ Rural

Smoking status: ☐ Yes ☐ No

Clinical Information

Presence of comorbidities: ☐ Yes ☐ No

If yes, specify: ____

Time since breast cancer diagnosis: ☐<1 year ☐ 1–3 years ☐>3 years

Type of surgery: ☐ Mastectomy ☐Breast conserving surgery☐ None

Previous endocrine and/or radiation therapy (before chemotherapy):

☐ Endocrine therapy only ☐ Radiation therapy only ☐ Both ☐ Neither

Section S2

Signed Informed Consent Form (English version)

**CONSENT FORM**<br>**to participate in the research study**	
**Please answer the following questionnaire in order to give written your participation consent to the following study:**	
**Depressive and Post-Traumatic Stress Symptoms and Their Associations with Health-Related Quality of Life in Women with Breast Cancer: A Cross-Sectional Secondary Analysis**	
Question	Yes or No
I have been told that this is an anonymous study that respects participants' identity and clinical data.	
I have received all the appropriate information about this study.	

I have been told that the authors currently plan to submit this study for publication in a medical journal, for educational purposes.	
I am not required to sign this form, and I may refuse to do so.	
I will not be paid in any manner to participate in this study.	
I finally give my permission to participate to the above-mentioned study.	

Surname (just the first letter):		Name:	
Signature:		Date:	

Supplementary Table S1. Reliability and summary results for the subdimensions of the QLQ-C30 scale, evaluating HRQoL.

	Mean	SD	Min	Max	95% Confidence Interval (lower/upper bound)	Reliability via Cronbach's alpha
**Global health status/QoL**	51.18	19.29	16.67	83.33	47.46/54.89	0.834
**Functional scales**						
Physical function	80.88	17.65	6.67	100.00	77.52/84.38	0.865
Rolefunction	57.39	25.73	0.00	100.00	52.63/62.60	0.882
Emotional function	51.10	28.80	0.00	100.00	45.11/56.15	0.809
Cognitive function	56.92	27.97	0.00	100.00	51.54/62.42	0.869
Social function	75.63	30.88	0.00	100.00	69.41/81.38	0.831
**Symptomscales/items**						
Fatigue	46.12	26.60	0.00	100.00	40.76/51.08	0.822
Nausea and vomiting	6.92	15.58	0.00	83.33	3.95/10.01	0.874
Pain	23.11	25.98	0.00	100.00	18.30/28.36	0.853
Dyspnea	21.70	23.92	0.00	100.00	17.27/26.53	0.813
Insomnia	32.70	29.09	0.00	100.00	27.04/38.35	0.882
Loss of appetite	12.89	19.83	0.00	100.00	9.16/16.89	0.806
Constipation	14.15	26.00	0.00	100.00	9.23/19.33	0.811
Diarrhea	3.17	9.83	0.00	33.33	1.27/5.07	0.828
Financial difficulties caused by physical condition or medical therapy	16.35	26.12	0.00	100.00	11.43/21.57	0.807

Note: SD: Standard deviation; Min: minimum value; Max: maximum value.

Supplementary table S2. Multiple regression analysis with total PHQ-9 score, evaluating depression, as a dependent variable.

	Unstandardized Coefficients		Standardized Coefficients			95.0% CI for B	
	B	SE	β	t-test	p-value	Lower Bound	Upper Bound
(Constant)	13.799	4.446		3.103	0.003*	4.964	22.634
Age (years)	0.060	0.045	0.144	1.321	0.190	-0.030	0.150
Employment status, reference: employed	1.324	1.248	0.108	1.061	0.292	-1.156	3.805
Education level, reference: Compulsory education							
Bachelor's degree	1.190	1.160	0.112	1.026	0.308	-1.115	3.495
Master's/PhD	1.785	1.421	0.133	1.256	0.212	-1.038	4.607
Marital status, reference: married							
Single	0.568	1.252	0.049	0.454	0.651	-1.919	3.055
Divorced	-2.140	1.329	-0.177	-1.610	0.111	-4.780	0.500
Widowed	-1.810	1.732	-0.108	-1.045	0.299	-5.251	1.631
Place of residence, reference: urban							
Suburban	-0.963	1.406	0-.087	-0.685	0.495	-3.757	1.831
Rural	-1.838	1.556	-0.161	-1.181	0.241	-4.930	1.254
CCI	-0.724	0.561	-0.134	-1.290	0.200	-1.839	0.391
Surgical treatment, reference: no mastectomy							
Mastectomy	2.416	1.055	0.245	2.289	0.024*	0.319	4.513
Type of treatment prior to chemotherapy, reference: Neither endocrine nor radiation therapy							
Endocrine therapy	-2.413	1.187	-0.217	-2.033	0.045*	-4.771	-0.055
Radiation therapy	-1.364	1.373	-0.118	-0.993	0.323	-4.093	1.365
Both	-2.175	1.448	-0.180	-1.502	0.137	-5.053	0.703
Time since BC diagnosis, reference: >3 years							
<1 year	1.479	1.640	0.124	0.902	0.370	-1.780	4.738
1-3 years	2.257	1.126	0.228	2.005	0.048*	0.020	4.495
$R^2 = 0.242$, adjusted $R^2 = 0.106$, F = 1.774, p = 0.047; Durbin–Watson = 2.116, VIFs = 1.205-2.201							

Note: B = unstandardized regression coefficient; SE = standard error; β = standardized regression coefficient; CI = confidence interval; CCI = Charlson Comorbidity Index; VIF: Variance Inflation Factor. *$p < 0.05$.

Supplementary table S3. Multiple regression analysis with total PCL-5 score, evaluating PTSD, as a dependent variable.

	Unstandardized Coefficients		Standardized Coefficients			95.0% CI for B	
	**B**	**SE**	**β**	**t-test**	**p-value**	**Lower Bound**	**Upper Bound**
(Constant)	0.089	13.866		0.006	0.995	-27.462	27.641
Age (years)	0-.068	0.141	-0.052	-0.484	0.630	-0.349	0.212
Employment status, reference: employed	-3.906	4.009	-0.101	-0.974	0.333	-11.871	4.060
Education level, reference: Compulsory education							
Bachelor's degree	4.503	3.725	0.136	1.209	0.230	-2.899	11.905
Master's/PhD	4.642	4.562	0.111	1.018	0.312	-4.422	13.707
Marital status, reference: married							
Single	-0.464	4.019	-0.013	-0.115	0.908	-8.450	7.523
Divorced	-0.304	4.267	-0.008	-0.071	0.943	-8.782	8.174
Widowed	-3.457	5.561	-0.066	-0.622	0.536	-14.507	7.593
Place of residence, reference: urban							
Suburban	-1.203	3.812	-0.035	-0.316	0.753	-8.777	6.370
Rural	12.758	4.651	0.337	2.743	0.007*	3.516	22.000
CCI	1.317	1.802	0.078	0.731	0.467	-2.263	4.897
Surgical treatment, reference: no mastectomy							
Mastectomy	7.134	3.389	0.232	2.105	0.038*	0.400	13.868
Type of treatment prior to chemotherapy, reference: Neither endocrine nor radiation therapy							
Endocrine therapy	-4.690	4.515	-0.136	-1.039	0.302	-13.661	4.281
Radiation therapy	2.874	4.997	0.081	0.575	0.567	-7.055	12.803
Both	-2.315	4.410	-0.064	-0.525	0.601	-11.078	6.448
Time since BC diagnosis, reference: >3 years							
<1 year	-5.835	5.087	-.156	-1.147	.254	-15.943	4.273
1-3 years	6.995	3.492	.226	2.003	.048	.058	13.933
R^2 = 0.247; adjusted R^2 = 0.112; F = 1.828; p = 0.039; Durbin–Watson = 1.569, VIFs = 1.205-2.201							

Note: B = unstandardized regression coefficient; SE = standard error; β = standardized regression coefficient; CI = confidence interval; CCI = Charlson Comorbidity Index; VIF: Variance Inflation Factor. *$p < 0.05$.

Supplementary Table S4. Associations between probable PTSD status and QoL dimensions (functioning and symptoms).

Dependent variable	Independent variable	Beta	Standard error	t	p	95.0% Confidence Interval for B	
						Lower Bound	Upper Bound
Global health status/QoL	Intercept	60.256	2.390	25.212	0.000	55.516	64.996
	PTSD	-17.646	3.364	-5.246	0.000	-24.318	-10.975
Physical function	Intercept	89.103	2.196	40.584	0.000	84.748	93.457
	PTSD	-16.147	3.090	-5.225	0.000	-22.275	-10.018
Role function	Intercept	69.231	3.205	21.601	0.000	62.875	75.587
	PTSD	-23.004	4.511	-5.100	0.000	-31.951	-14.058
Emotional function	Intercept	58.814	3.810	15.436	0.000	51.257	66.371
	PTSD	-16.204	5.363	-3.021	0.003	-26.840	-5.568
Cognitive function	Intercept	66.987	3.661	18.297	0.000	59.726	74.248
	PTSD	-19.817	5.153	-3.846	0.000	-30.037	-9.597
Social function	Intercept	84.295	4.130	20.412	0.000	76.105	92.485
	PTSD	-17.628	5.813	-3.033	0.003	-29.156	-6.100
Fatigue	Intercept	32.265	3.193	10.104	0.000	25.932	38.598
	PTSD	27.064	4.495	6.021	0.000	18.150	35.978
Nausea and vomiting	Intercept	2.885	2.104	1.371	0.173	-1.288	7.057
	PTSD	8.122	2.961	2.743	0.007	2.249	13.994
Pain	Intercept	14.423	3.407	4.234	0.000	7.667	21.179
	PTSD	17.652	4.795	3.682	0.000	8.143	27.162
Dyspnoea	Intercept	14.744	3.185	4.629	0.000	8.427	21.060
	PTSD	14.187	4.483	3.165	0.002	5.296	23.078
Insomnia	Intercept	23.077	3.848	5.997	0.000	15.445	30.709
	PTSD	19.061	5.416	3.519	0.001	8.320	29.803
Appetite loss	Intercept	7.692	2.670	2.881	0.005	2.397	12.988
	PTSD	10.547	3.758	2.806	0.006	3.094	18.000
Constipation	Intercept	9.615	3.577	2.688	.008	2.520	16.710
	PTSD	9.253	5.035	1.838	0.069	-.734	19.239
Diarrhea	Intercept	2.564	1.367	1.875	0.064	-.148	5.276
	PTSD	1.209	1.925	.628	0.531	-2.608	5.027
Financial difficulties caused by physical condition or medical treatment	Intercept	10.897	3.567	3.055	0.003	3.823	17.972
	PTSD	11.115	5.021	2.214	0.029	1.158	21.072

Note: PTSD: Post-traumatic stress disorder; *Significant at the level of p<0.05.

Supplementary Table S5. Associations between depressive symptom severity and QoL dimensions (functioning and symptoms).

Dependent variable	Independent variable	Beta	Standard error	t	p	95.0% Confidence Interval for B

						Lower Bound	Upper Bound
Global health status/QoL	Intercept	77.613	4.105	18.905	0.000*	69.471	85.755
	PHQ-9	-2.196	.317	-6.919	0.000*	-2.825	-1.566
Physical function	Intercept	107.779	3.553	30.332	0.000*	100.731	114.826
	PHQ-9	-2.243	.275	-8.166	0.000*	-2.787	-1.698
Role function	Intercept	95.055	5.288	17.975	0.000*	84.567	105.543
	PHQ-9	-3.130	.409	-7.657	0.000*	-3.940	-2.319
Emotional function	Intercept	79.373	6.673	11.895	0.000*	66.139	92.608
	PHQ-9	-2.402	.516	-4.658	0.000*	-3.425	-1.380
Cognitive function	Intercept	84.822	6.594	12.863	0.000*	71.744	97.900
	PHQ-9	-2.327	.510	-4.566	0.000*	-3.338	-1.316
Social function	Intercept	115.312	6.728	17.138	0.000*	101.968	128.656
	PHQ-9	-3.337	.520	-6.416	0.000*	-4.368	-2.305
Fatigue	Intercept	14.179	5.965	2.377	0.019*	2.349	26.009
	PHQ-9	2.654	.461	5.757	0.000*	1.740	3.568
Nausea and vomiting	Intercept	-5.334	3.804	-1.402	0.164	-12.880	2.211
	PHQ-9	1.030	.294	3.502	0.001*	.447	1.613
Pain	Intercept	-10.776	5.619	-1.918	0.058	-21.921	.368
	PHQ-9	2.852	.434	6.565	0.000*	1.990	3.713
Dyspnoea	Intercept	-8.663	5.230	-1.656	0.101	-19.036	1.711
	PHQ-9	2.555	.404	6.321	0.000*	1.754	3.357
Insomnia	Intercept	4.429	6.892	.643	0.522	-9.241	18.098
	PHQ-9	2.363	.533	4.436	0.000*	1.307	3.420
Appetite loss	Intercept	-4.416	4.767	-.926	0.356	-13.870	5.038
	PHQ-9	1.457	.368	3.955	0.000*	.727	2.188
Constipation	Intercept	6.362	6.661	.955	0.342	-6.849	19.572
	PHQ-9	.662	.515	1.287	0.201	-.359	1.684
Diarrhea	Intercept	.615	2.516	.245	0.807	-4.374	5.604
	PHQ-9	.214	.194	1.101	0.274	-.172	.600
Financial difficulties caused by physical condition or medical treatment	Intercept	-3.958	6.380	-.620	0.536	-16.611	8.695
	PHQ-9	1.711	.493	3.470	0.001*	.733	2.689

Note: PTSD: Post-traumatic stress disorder; *Significant at the level of p<0.05.
